# Research on microbial communities in tumor microenvironments: cutting-edge dynamics and future trends from a bibliometric perspective

**DOI:** 10.3389/fimmu.2026.1745842

**Published:** 2026-02-16

**Authors:** Xuan Tang, Changfeng Man, Ziyan Liu, Dandan Gong, Yu Fan

**Affiliations:** Cancer Institute, Affiliated People’s Hospital of Jiangsu University, Zhenjiang, Jiangsu, China

**Keywords:** bibliometric analysis, cancer therapy, Fusobacterium nucleatum, immune microenvironment, intratumoral microbiota

## Abstract

**Background:**

In recent years, researchers have identified numerous potential biomarkers and therapeutic targets applicable to cancer immunotherapy, among which the role of tumor-internal microorganisms in the tumor microenvironment has been explored. However, this field is still in its early stages of development, facing limitations such as the unclear mechanisms of interaction between tumor-internal microorganisms and host immunity, as well as significant variations in microbial profiles among different tumor types and patients.

**Objective:**

This study aims to explore the research hotspots and development trends of tumor-internal microorganisms through bibliometric methods and to construct a systematic knowledge map.

**Methods:**

This study retrieved publications related to tumor-internal microorganisms from the Web of Science Core Collection (WOSCC) prior to December 22, 2025. Subsequently, the selected literature was analyzed using VOSviewer (v.1.6.20), CiteSpace (v.6.4.1R), and SCImago Graphica. In addition, we integrated PubMed data to assess status and trends in preclinical and clinical studies of intratumoral microbiota interventions for anti-tumor therapy efficacy.

**Results:**

From the Web of Science database, we retrieved 1,278 relevant articles. Since 2012, the number of papers published on the intratumoral microbiota has shown an overall upward trend. China and the United States are the two major countries in this field. Keyword analysis shows that “tumor microbiome,” “gut microbiome,” “cancer,” and “*Fusobacterium nucleatum*” are frequent terms. 11 keyword groups have been identified, among which “tumor immunotherapy” and “immune microenvironment” form two important groups. A total of 69 preclinical and clinical studies has intervened in intratumoral microbiota and affected anti-tumor treatment outcomes. Among them, 25 studies involving *Fusobacterium nucleatum* account for a large proportion. However, most of these studies are still at the basic or preclinical stage, and clinical translation evidence is limited.

## 1 Introduction

In recent years, research on the tumor microenvironment (TME) has deepened, and in addition to the traditional focus on tumor cells, immune cells, and stromal cells, the intratumoral microbiome (ITM) has gradually emerged as a new direction in oncology research (1–3). Since its systematic reporting in 2020 as being present in cancer cells and immune cells (4), numerous studies have discovered that intratumoral microbes not only exhibit high heterogeneity (5) and low biomass characteristics (6, 7) within tumor tissues but may also play significant roles in tumorigenesis, progression, recurrence, and prognosis by influencing processes (8–10)such as tumor inflammatory responses (11), immune evasion (12), genomic stability (13), and treatment resistance (14). Furthermore, significant differences in the composition of intratumoral microbiota exist among patients of different tumor origins, subtypes (15, 16), ethnicities (17), and lifestyles (18), suggesting that intratumoral microbes hold promise as potential biomarkers and therapeutic targets in cancer diagnosis and treatment.

In terms of detection technology, the development of new methods such as 2bRAD-M sequencing, 16S rRNA sequencing, and spatial transcriptomics has significantly enhanced the detection and functional analysis capabilities of low-abundance microbes, providing powerful tools for elucidating the biological significance of intratumoral microbiota (19–21). In clinical application explorations, existing research indicates that intratumoral microbes can modulate the efficacy of immunotherapy, targeted therapy, chemotherapy, and radiotherapy (22–25). Some studies have even combined antimicrobial therapy with nanocarrier technology to improve microbe-related immune suppression (26, 27). However, this field still faces numerous challenges, including high risks of sample contamination, lack of standardized detection methods, insufficient mechanistic studies, and the complexity of interactions among different microbial communities, which to some extent limits the reproducibility of research results and their clinical translatability.

Against this backdrop, it is essential to systematically review and quantitatively analyze the knowledge structure, hot topics, cutting-edge trends, and academic collaboration networks in the field of intratumoral microbiota research. Bibliometrics, as a method that combines quantitative statistics and visual analysis, can reveal the development history, core scholars, major research institutions, key literature, and future directions of specific research fields, and has been widely applied in the analysis of research hotspots in oncology and microbiology (28). Therefore, this study aims to conduct a systematic analysis of literature related to intratumoral microbiota based on the internationally recognized database Web of Science, utilizing bibliometric and visualization techniques, with the goal of mapping the overall research landscape of this field, identifying its core research hotspots and potential cutting-edge developments, and providing references for subsequent basic research, clinical translation, and interdisciplinary collaboration.

## 2 Materials and methods

### 2.1 Sources of literature and search strategy

**Figure 1:**
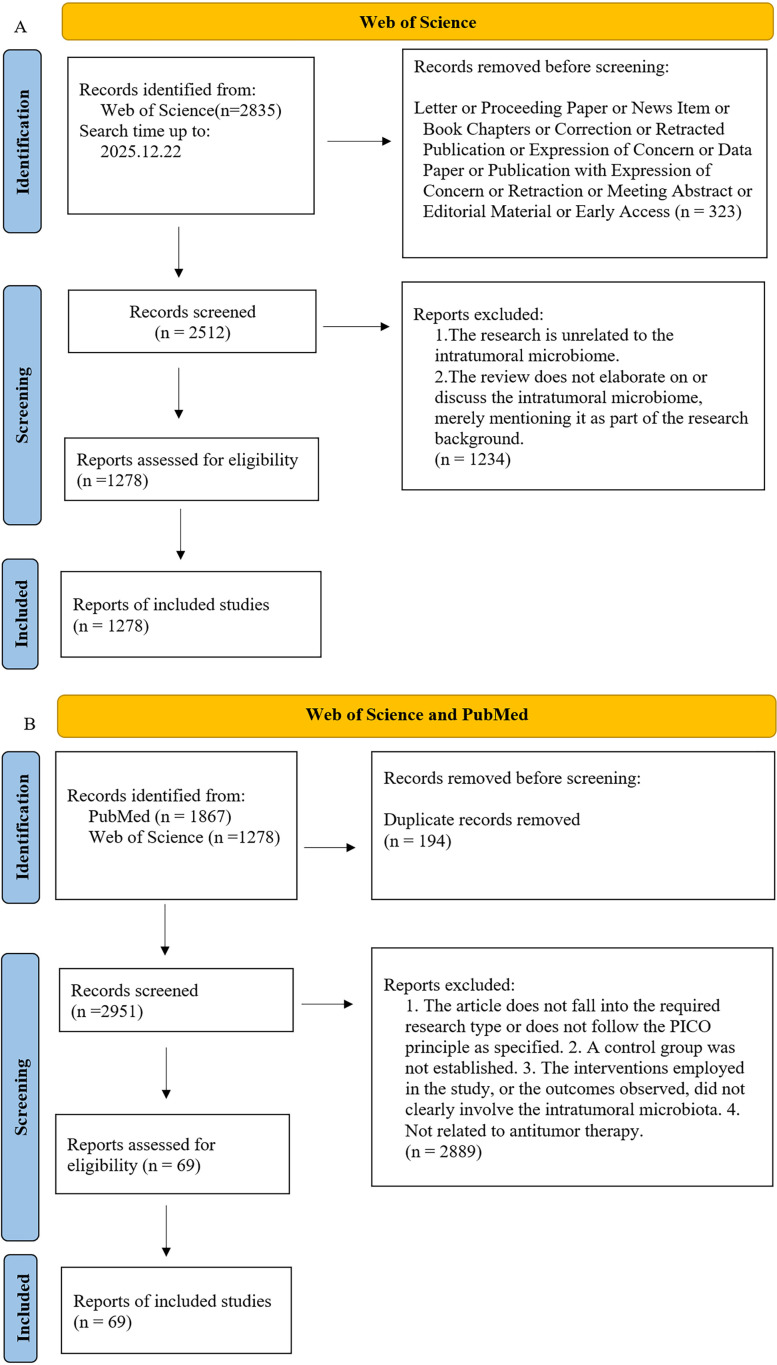
Flow chart of literature retrieval. **(A)** Flowchart of the Web of Science search process, with search results incorporated into software for visualizing bibliometric analysis. **(B)** Flowchart of the Web of Science and PubMed search processes, with search results analyzed for the status and trends of preclinical and clinical trial research. The flowchart was created using the PRISMA 2020 flow diagram for updated systematic reviews, available at: https://www.prisma-statement.org/prisma-2020-flow-diagram.

In this study, the literature search mainly came from two databases: Web of Science and PubMed, covering relevant research from the establishment of the database until December 22, 2025. We conducted a systematic search for the subject term “intratumoral microbiota,” with specific search strategies outlined in the “**Supplementary File 1**.” Through the search in Web of Science, we initially identified 2,835 records. After screening, a total of 1,278 documents were included, forming the bibliometric dataset used for visual analysis of annual publication volume, co-authorship, co-citation, and keyword mapping, with the specific search process detailed in **Figure 1A** and “**Supplementary File 1**.” In addition, 1,867 records were retrieved from the PubMed database. After removing duplicates in comparison with the 1,278 documents included from Web of Science, the total number of documents from both Web of Science and PubMed was 2,951. Subsequently, we excluded 2,886 documents that did not meet the research criteria, resulting in a final number of 69 documents that met the inclusion criteria. Detailed information on the search process can be found in **Figure 1B** and the “**Supplementary File 1**.” These 69 documents will be used for subsequent analysis of trends in preclinical research and clinical trial studies. The retrieval and screening process is shown in **Figure 1**:

### 2.2 Inclusion and exclusion criteria

Eligible studies include original research and review articles that directly investigate the relationship between intratumoral microbiota and tumors. Exclusion criteria encompass non-research materials (such as editorials, book chapters, and conference abstracts), duplicate entries, retracted publications, and studies with insufficient or incomplete data. Additionally, studies unrelated to intratumoral microbiota were also manually excluded. Two researchers independently conducted the screening, data extraction, and verification, resolving any discrepancies through team discussions. The final dataset includes complete records of Full Record.

The literature on preclinical and clinical studies of intratumoral microbiota interventions for anti-tumor therapy efficacy applied stringent inclusion and exclusion criteria. Inclusion criteria: 1. Research type: Clinical Study, Clinical Trial, Clinical Trial, Phase I, Clinical Trial, Phase II, Clinical Trial, Phase III, Clinical Trial, Phase IV, Controlled Clinical Trial, Pragmatic Clinical Trial, Randomized Controlled Trial. 2. PICO principles: P (Participant): Tumor patients, tumor cells, or experimental animals; no species restrictions. I (Intervention): Interventions targeting naturally occurring intratumoral microbiota to influence anti-tumor treatment efficacy. C (Comparison): Blank control group or placebo group. O (Outcome): Tumor treatment efficacy. Exclusion criteria: 1. A control group was not established. 2. The interventions employed in the study, or the outcomes observed, did not clearly involve the intratumoral microbiota. 3. Not related to Anti-tumor therapy.

### 2.3 Data analysis and visualization

This study employed four software tools for bibliometric and visualization analysis, including Microsoft Excel 2022, VOSviewer (v.1.6.20), CiteSpace (v6.4.R1), and SCImago Graphica.

#### 2.3.1 VOSviewer (v.1.6.20)

It was used to generate network diagrams of country/regional cooperation (**Figure 3A**), institutional cooperation network (**Figure 4B**), and author cooperation network (**Figure 5**). The analysis adopted the “total count” method built into the software. To focus on core participants, the minimum publication volume threshold was set as follows: authors ≥ 6, countries/regions ≥ 8, institutions ≥ 11. In the network diagrams, the size of the nodes is proportional to the number of publications, the color represents different clusters, and the thickness of the lines reflects the intensity of cooperation.

**Figure 2:**
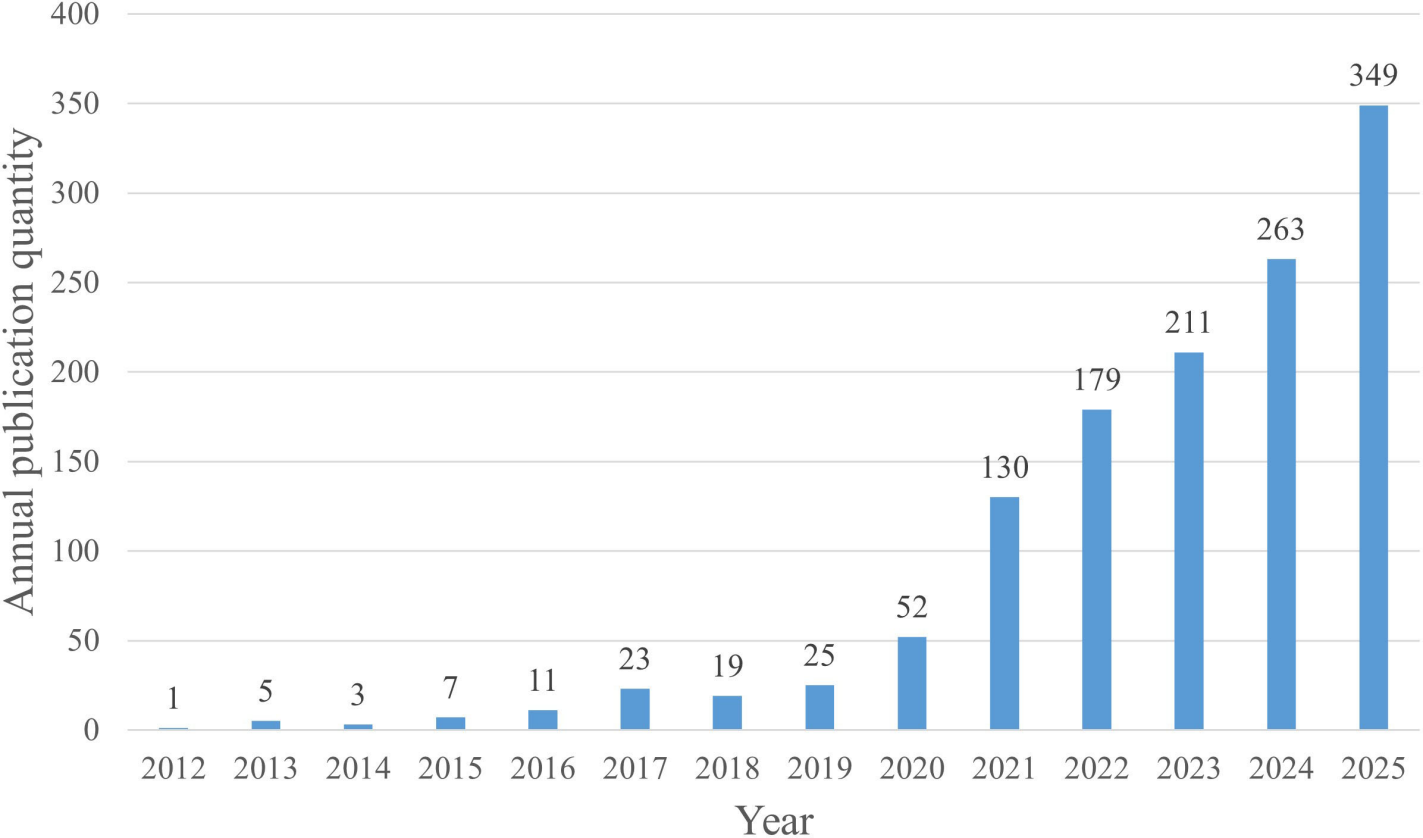
Overview of intratumoral microorganism–related publications by volume. Annual publication numbers from 2012 to 2025.

**Figure 3:**
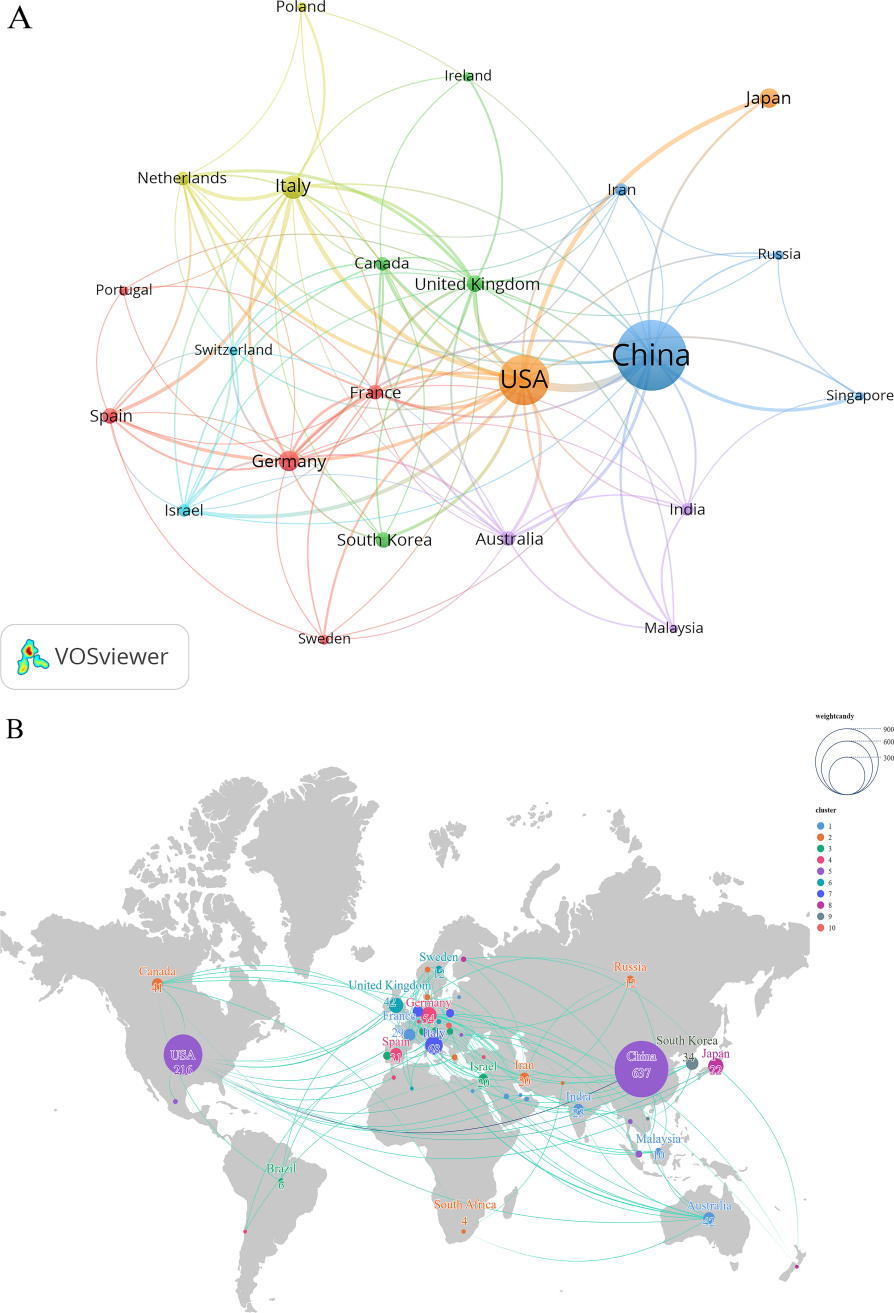
Participation of countries/regions in intratumoral microbiome research. **(A)** A network diagram assessing global collaboration. **(B)** A world map of intratumoral microbiomes. The numerical values correspond to the publication volume of each country, the text denotes the names of the countries, the size of the circles reflects the proportion of publication volume, and the lines indicate collaborations between different countries.

**Figure 4:**
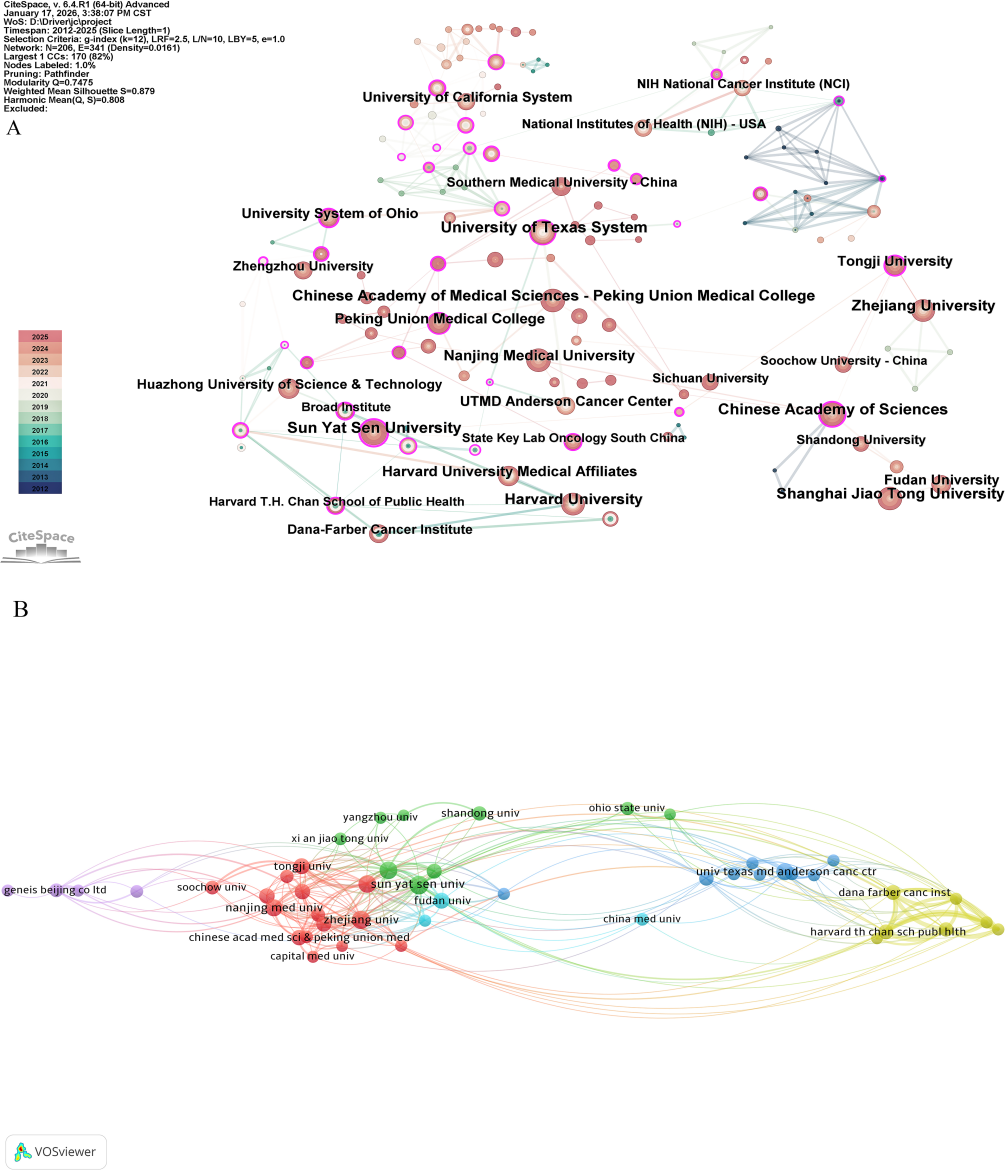
Visualization of institutions related to intratumoral microbiome research: institutional co-occurrence network view.

#### 2.3.2 CiteSpace (v6.4.R1)

CiteSpace (v6.4.R1) was used for institution cooperation network analysis (**Figure 4A**), keyword co-occurrence (**Figure 6**), clustering (**Figure 7**), emergent (**Figure 8**), and timeline evolution (**Figure 9**) analysis. The parameters were set as follows: time slice set to 1 year, analysis range up to 2025. The node types included institutions, authors, and keywords. The network was optimized by using the Pathfinder, Pruning sliced network, and Pruning the merged network algorithms. The main network parameters were: g-index (k=25), node number (N)=272, edge number (E)=418, network density (Density)=0.0113. The settings for emergent word detection were γ (1.0), minimum duration = 1. The specific details of keyword merging can be found in the appendix “**Supplementary materials**.”.

**Figure 5:**
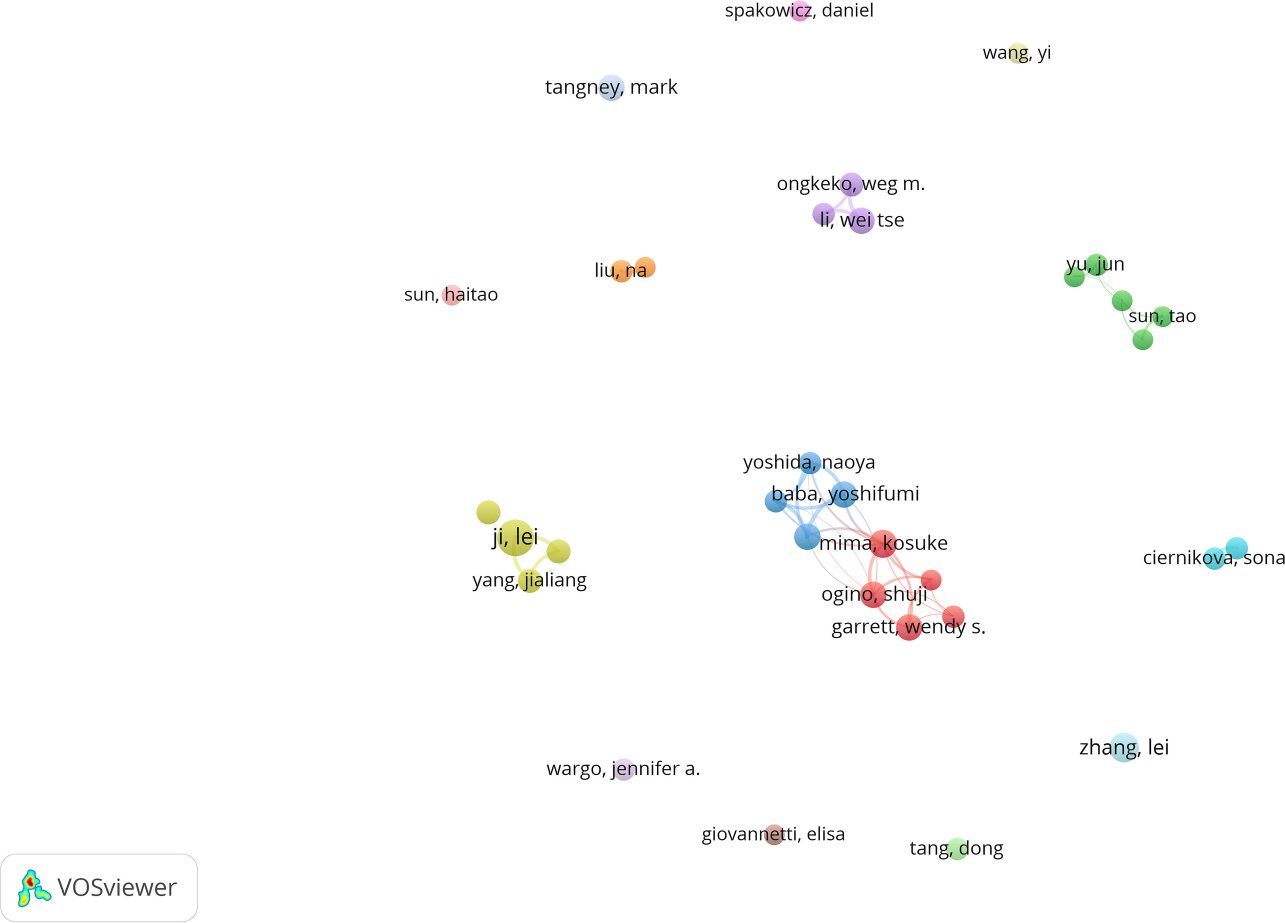
Visual analysis of publishing authors. The cooperation network of co-occurring authors visualized using VOSviewer.

**Figure 6:**
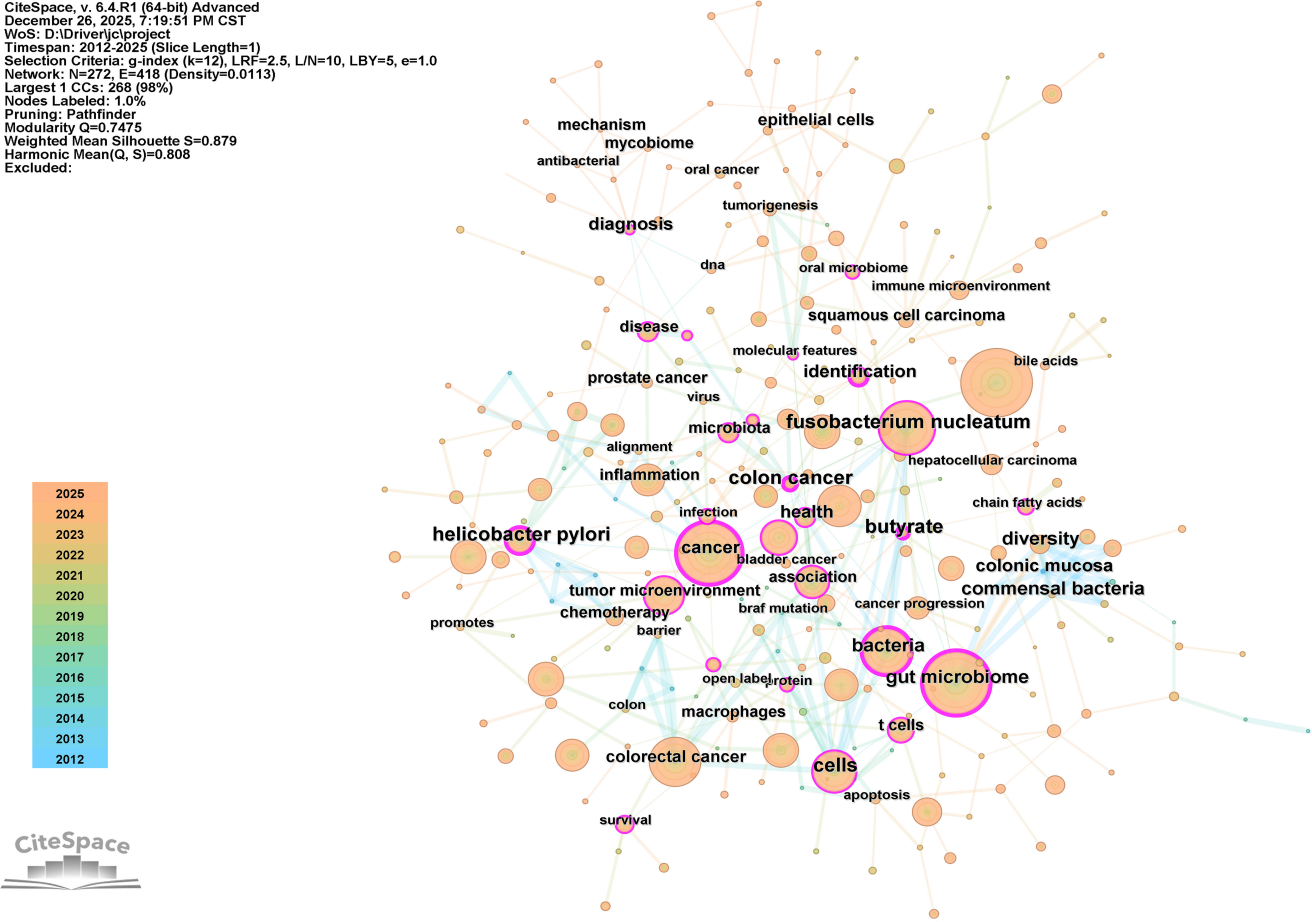
A representation of the keyword mapping focused on intratumoral microbiomes. The shade of the node reflects the frequency of occurrence.

**Figure 7:**
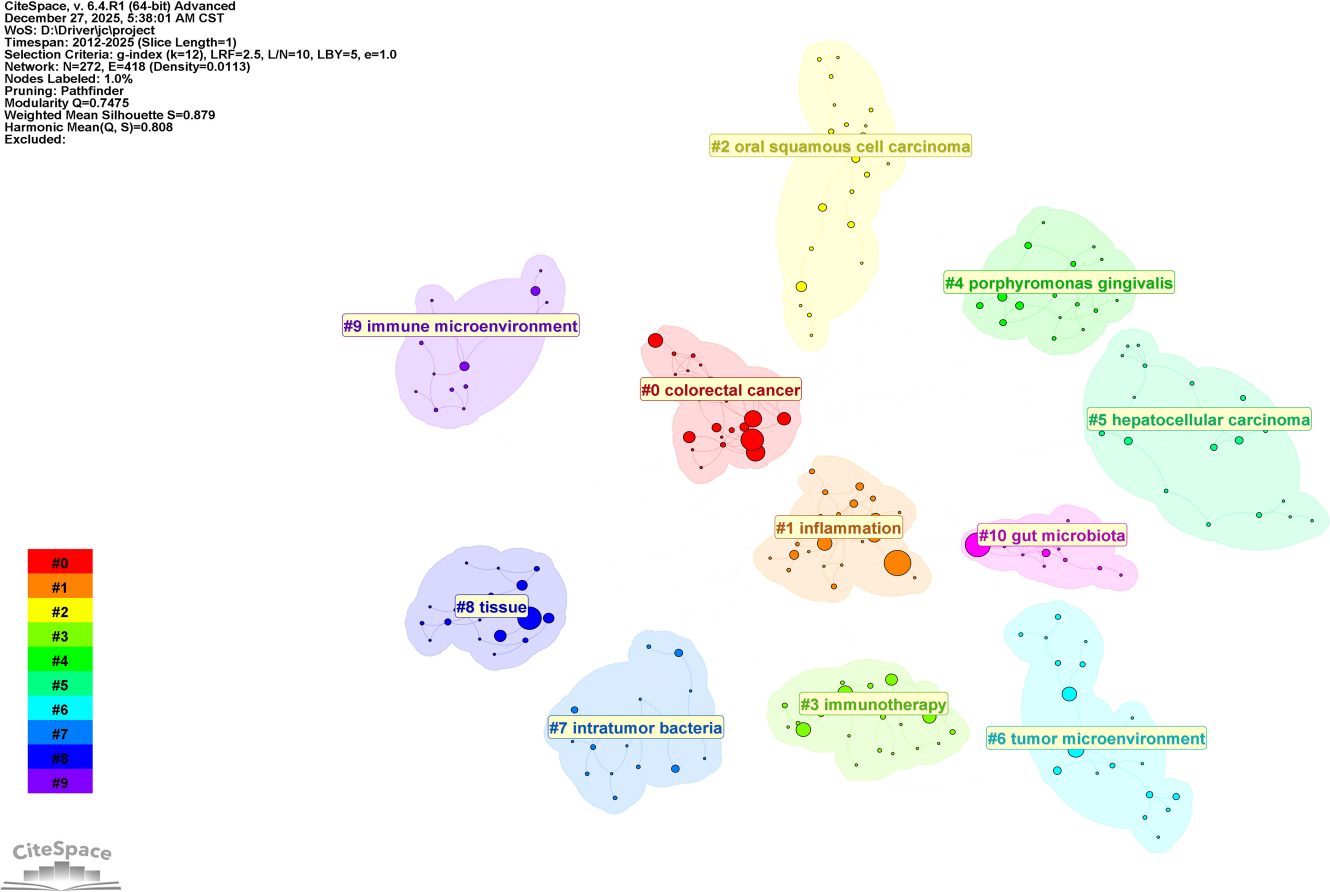
Network map of keyword clustering for intratumoral microbiomes.

**Figure 8:**
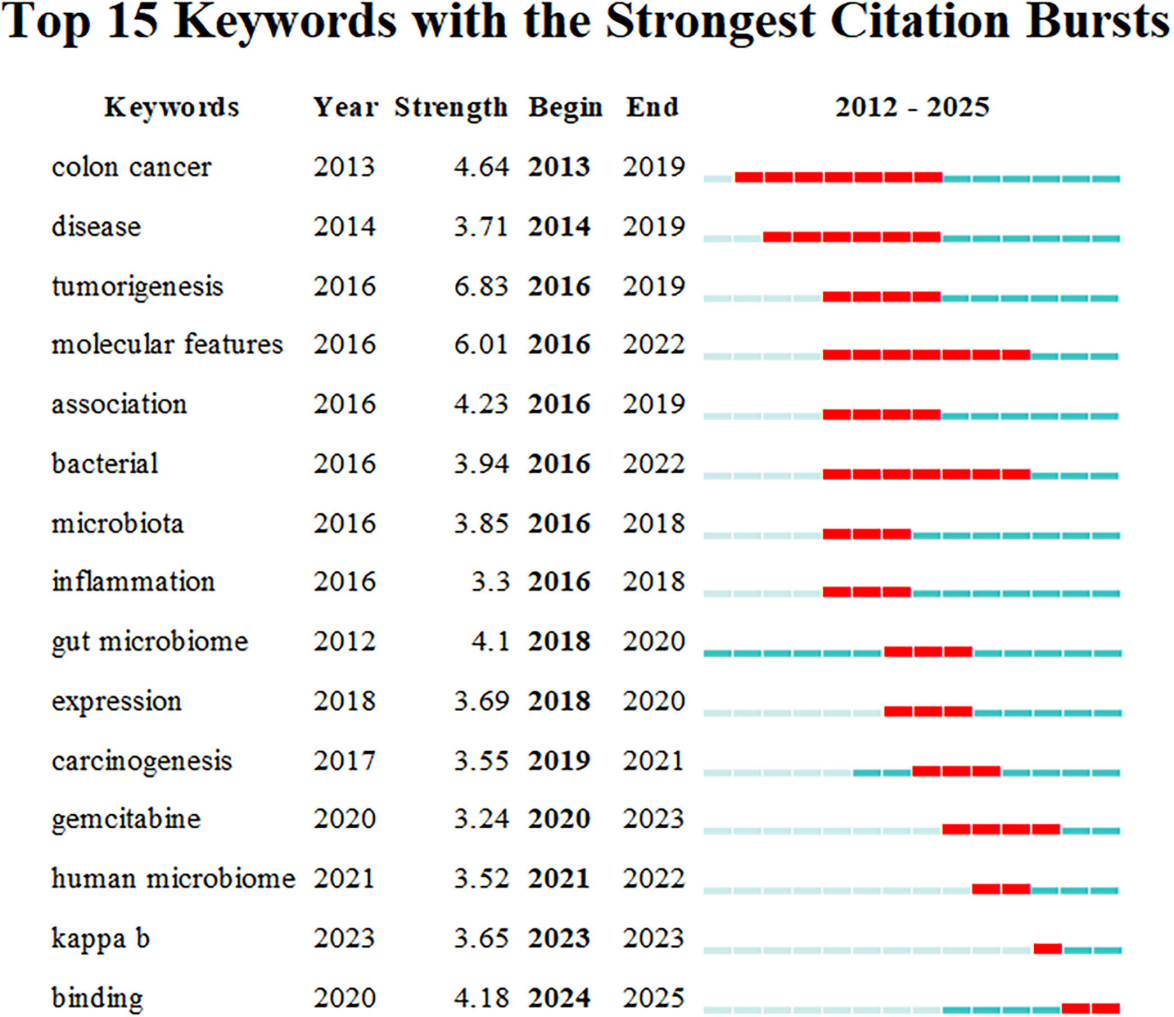
The top 15 keywords with the strongest citation burst. The blue bars represent the time periods during which the keywords appeared, while the red bars indicate the intervals in which citation bursts were observed, denoting the starting year, ending year, and duration of the bursts.

#### 2.3.3 SCImago Graphica

SCImago Graphica was used to convert the publication volume and cooperation data of countries/regions into geographic space visualization graphics (**Figure 3B**). Firstly, the country cooperation matrix data (in CSV format) was exported from VOSviewer, and then the results were imported into SCImago Graphica for visualization analysis. The size of the circles in the figure is proportional to the publication volume, the numbers correspond to the publication volume of the countries, and the lines represent the cooperative relationships between countries/regions. The thickness of the lines represents the number of collaborations.

#### 2.3.4 Microsoft Excel 2022

Microsoft Excel 2022 was used for basic data organization and the drawing of descriptive statistical charts, namely the annual publication volume trend chart (**Figure 2**).

**Figure 9:**
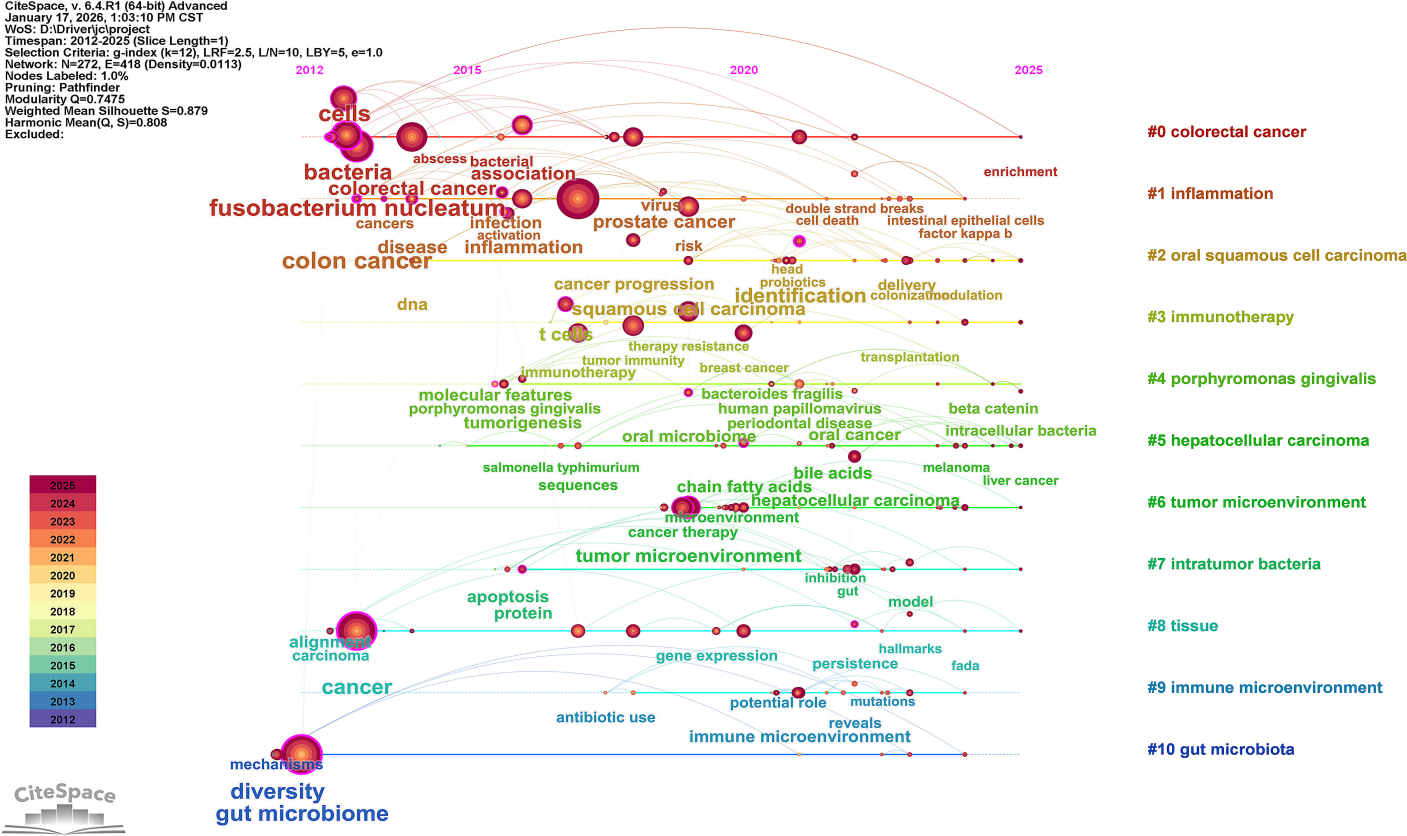
Timeline of the keyword clustering network map for intratumoral microbiomes.

## 3 Result

### 3.1 Literature search results

This study incorporated 1,278 eligible documents from the Web of Science for visual analysis. The Web of Science records show that the earliest study addressing the relationship between intratumoral microbiomes and tumors was published in 2012. From 2012 through 2025, annual publication counts in this field have generally increased, with a dip in 2018 and subsequent year-on-year growth from 2019 onward, accompanied by a rapid rise after 2020 (**Figure 2**), indicating that this field has attracted sustained and rapidly growing research interest in recent years. Notably, in 2020, Nejman et al. proposed that different cancer types exhibit distinct intratumoral microbiome compositions and that most intratumoral bacteria reside within cancer and immune cells (4), a finding that coincided with a surge in publications thereafter. This development has prompted many researchers to shift their focus from merely detecting microbiomes in tumor tissues to conducting *in vivo* and *in vitro* experiments on microbiomes within tumors and immune cells. In 2022, a Nature study combined 10x Visium spatial transcriptomics with GeoMx digital spatial analysis, integrating *in situ* spatial analysis with single-cell RNA sequencing (29), and this work revealed interactions between the host and microbiome at spatial and cellular levels. These technological advances have propelled multi-omics studies of intratumoral microbiomes, further shifting the research emphasis from simply characterizing microbiome composition to exploring function and potential mechanisms. It is important to note that the publication count for 2025 is based on data available through December 22, 2025, and may not fully reflect the final total for that year (**Figure 2**).

### 3.2 Distribution and collaboration among countries/regions.

**Figure 3** presents a map of publication volume and co-occurrence, depicting the countries and regions engaged in intratumoral microbiome research. The bibliometric analysis employed the Full Counting method available in VOSviewer software. Since the inception of the Web of Science database, a total of 69 countries and regions have contributed to this research field. **Table 1** lists the top ten countries and regions ranked by the number of published papers, with China leading at 637 publications, followed by the United States (330 papers), Italy (68 papers), Germany (54 papers), Japan (51 papers), and the United Kingdom (42 papers). In terms of citation frequency, the United States ranks first with a total of 26,772 citations, surpassing China’s 13,592 citations. France, Spain, and Italy also exhibit notable citation performance despite their relatively limited publication volumes. The analysis of national collaboration networks indicates that a close collaborative structure has formed globally, centered around a few high-output countries. Among them, China and the United States constitute dual hubs of the network, with the strongest collaborative link between the two. Additionally, the United States maintains a stable cooperative relationship with major European countries such as Germany and the United Kingdom, while there is also active regional collaboration among European countries, resulting in a tight-knit sub-network. Overall, the field of intratumoral microbiome research demonstrates a broadly international collaborative landscape.

**Table 1:** Top 10 countries/regions by publication output.

Rank	Country	Documents	Citations
1	China	637	13592
2	USA	330	26772
3	Italy	68	3670
4	Germany	54	2392
5	Japan	51	2322
6	United Kingdom	42	2025
7	South Korea	34	895
8	Spain	31	2504
9	Australia	29	1662
10	France	29	2804

### 3.3 Analysis of collaborative relationships among publishing institutions

The VOSviewer parameters were set as Method (Linlog/modularity) and a minimum number of documents from an institution: 11. The obtained results were retrieved from 1,935 institutions, and 47 met the thresholds. As shown in **Figure 4B**, six clusters were formed, each comprising institutions that collaborate closely. The first cluster includes the following institutions: Capital Medical University; Central South University; Chinese Academy of Medical Sciences; Chinese Academy of Medical Sciences & Peking Union Medical College; Huazhong University of Science and Technology; Nanjing Medical University; Peking University; Shanghai Jiao Tong University; Soochow University; Southern Medical University; Tongji University; Wuhan University; Zhejiang Chinese Medical University; Zhejiang University; and Zhengzhou University. The second cluster consists of the following institutions: Chinese Academy of Sciences; Mayo Clinic; Ohio State University; Qingdao University; Shandong University; Sichuan University; Sun Yat-sen University; Xi’an Jiaotong University; and Yangzhou University. The third cluster includes the following institutions: Baylor College of Medicine; Cleveland Clinic; Johns Hopkins University; Nanchang University; National Cancer Institute; University of California San Diego; University of Maryland; University of Texas MD Anderson Cancer Center; and Weizmann Institute of Science. The fourth cluster comprises the following institutions: Brigham and Women’s Hospital; Broad Institute of MIT and Harvard; Dana-Farber Cancer Institute; Harvard Medical School; Harvard T.H. Chan School of Public Health; Kumamoto University; and Massachusetts General Hospital. The fifth cluster includes the following institutions: Fujian Medical University; Geneis Beijing Co., Ltd.; Harbin Medical University; and Qingdao Geneis Institute of Big Data and Precision Medicine. The sixth cluster consists of China Medical University; Fudan University; and the University of Hong Kong. **Figure 4A** displays a visualization map of the CiteSpace network of institutions. We can observe that the connections between institutions are relatively close, with the outer purple and larger nodes representing significant participating institutions in intratumoral microbiome research. **Table 2** lists the publication volume and betweenness centrality of institutions, with Sun Yat-sen University ranking first in both metrics (46 papers and 0.41, respectively), indicating that it serves as a key bridge in the inter-institutional collaboration network.

**Table 2:** Top 10 institutions in terms of publications.

Rank	Organization	Documents	Centrality
1	Sun Yat Sen University	46	0.41
2	Chinese Academy of Sciences	45	0.24
3	University of Texas System	42	0.18
4	Harvard University	40	0.01
5	Chinese Academy of Medical Sciences - Peking Union Medical College	39	0.02
6	Zhejiang University	39	0.05
7	Shanghai Jiao Tong University	38	0
8	Nanjing Medical University	36	0.09
9	Harvard University Medical Affiliates	33	0.03
10	Peking Union Medical College	30	0.15

### 3.4 Author analysis

**Figure 5** illustrates the authors’ contributions and connections to the study of the intratumoral microbiome. **Table 3** presents the top ten most prolific and influential authors in this research field. The results indicate that research in this area is not concentrated within a single institution or author. In terms of publication volume, Ji Lei ranks first with 16 papers, demonstrating his sustained productivity in intratumoral microbiome research. His studies primarily focus on the associations between intratumoral microbiomes in gastrointestinal tumors and clinical outcomes, treatment responses, and immune-related indicators. Furthermore, Mima, Kosuke, Baba, Hideo, and Baba, Yoshifumi are also among the top authors in terms of publication volume and citation frequency, all maintaining close collaborative ties with Kumamoto University in Japan, forming a persistent and stable research cluster in this field. Notably, Baba, Hideo’s research focuses on the tissue detection of intratumoral Fusobacterium nucleatum and its clinical association with patient prognosis; Mima, Kosuke systematically analyzes the relationship between intratumoral microbiomes and the characteristics of the tumor immune microenvironment, advancing the direction from simple prognostic associations to immune phenotype analysis. Baba, Yoshifumi’s core contribution lies in the establishment of a systematic research framework centered on *Fusobacterium nucleatum*, integrating clinical outcomes, immune microenvironment, epigenetic changes, metabolic states, and molecular mechanisms. Although Garrett, Wendy S., and Bullman, Susan have published fewer articles, they rank first and third in citation frequency. Bullman, Susan’s work relies on a large prospective cohort and multidimensional data integration, focusing on revealing the heterogeneous contributions of intratumoral microbiomes in different subtypes of colorectal cancer, tumor locations, autophagy status, and treatment responses, particularly emphasizing the potential application value of intratumoral microbiomes in tumor.

**Table 3:** Top 10 authors by number of publications and citations.

Rank	Author	Number of publications	Author	Citations
1	Ji, Lei	16	Garrett, Wendy s.	2492
2	Zhang, Lei	11	Wargo, Jennifer a.	1703
3	Mima, Kosuke	10	Bullman, Susan	1421
4	Baba, Hideo	9	Mima, Kosuke	1299
5	Baba, Yoshifumi	9	Ogino, Shuji	905
6	Garrett, Wendy s.	9	Chan, Andrew t.	8281
7	Li, Wei tse	9	Baba, Hideo	765
8	Ogino, Shuji	9	Baba, Yoshifumi	765
9	Tangney, Mark	9	Yoshida, Naoya	723
10	Ongkeko, Weg m.	8	Tangney, Mark	643

### 3.5 Keyword analysis

Keywords serve as concise indicators of the central theme of research papers, capturing the essence of research questions and significant findings. They provide a brief overview of the research topic and its contributions to the academic field. Analyzing these keywords can identify key research hotspots, emerging trends, and areas of interest within the academic community. **Figure 6** visually illustrates the most used keywords, aiding in the identification of focal areas and overall trends in the study of the intratumoral microbiome. Additionally, **Table 4** organizes and lists the top ten keywords based on their frequency and betweenness centrality. Terms such as “ *tumor microbiome*,” “*gut microbiome*,” “*cancer*,” “*Fusobacterium nucleatum*,” and “*colorectal cancer*” hold significant positions, underscoring their relevance in ongoing discussions.

**Table 4:** The top ten keywords with the highest frequency (left) and the top ten keywords with the highest betweeness centrality (right).

Rank	Keyword	Counts	Centrality	Rank	Keywords	Counts	Centrality
1	tumor microbiome	392	0.01	1	colon cancer	27	0.35
2	gut microbiome	353	0.25	2	cancer	318	0.29
3	cancer	318	0.29	3	gut microbiome	353	0.25
4	*Fusobacterium nucleatum*	269	0.19	4	butyrate	11	0.25
5	colorectal cancer	244	0.07	5	bacteria	179	0.24
6	bacteria	179	0.24	6	identification	26	0.23
7	cells	150	0.12	7	helicobacter pylori	72	0.21
8	intratumor microbiome	137	0.02	8	health	46	0.2
9	tumor microenvironment	129	0.13	9	*Fusobacterium nucleatum*	269	0.19
10	tumor immunity	117	0.02	10	association	95	0.16

**Figure 7** illustrates the clustering of keywords, revealing 11 distinct clusters. Clustering employs the LLR clustering labeling method, resulting in Q = 0.7475 and S = 0.879. **Table 5** presents the cluster labels and the primary keywords for each cluster, including#0 *colorectal cancer*; #1 *inflammation*; #2 *oral squamous cell carcinoma*; #3 *immunotherapy*; #4 *porphyromonas gingivalis*; #5 *hepatocellular carcinoma*; #6 *tumor microenvironment*; #7 *intratumor bacteria*; #8 *tissue;* #9 *immune microenvironment*; #10 *gut microbiota*. The analysis based on keyword clustering indicates that research related to the intratumoral microbiome can be categorized into several relatively focused research groups. Each cluster exhibits a certain degree of internal consistency in keyword composition; however, some overlap exists between different clusters, suggesting a strong interrelation among research themes in this field. One cluster features high-frequency keywords such as *tumor microenvironment*, *tumor microbiota*, and *intratumoral microbiota* (#6), co-occurring with terms like *toll-like receptor*, *host microbial interactions*, and *breast cancer*, indicating that this cluster primarily consists of keywords describing the local tumor microenvironment and its associated microbial elements. Another cluster centers around core keywords like” *colorectal cancer* “ and” *fusobacterium nucleatum*” (#0, #1, and #4), appearing alongside terms such as *tumor microbiome*, *tumorigenesis*, and *poor prognosis*, reflecting that this line of research largely revolves around tumor-associated microbiota and their biological characteristics within the context of specific cancer types. Additionally, some clusters focus on keywords related to “ *immunotherapy*”, “ *immune microenvironment*”, and “ *immune checkpoint inhibitors* “ (#3 and #9), demonstrating a notable co-occurrence between the study of intratumoral microbiota and themes related to tumor immunotherapy. Furthermore, there are clusters concerning *intratumor bacteria* (#7) and *gut microbiota* (#10), which respectively examine the presence of bacteria within tumor tissues and the relationship between gut microbiota and tumor immunotherapy research. Overall, the results of the keyword clustering reflect the diversity of current research on intratumoral microbiota in terms of subjects and content; however, the different themes do not form completely isolated structures but rather exhibit a degree of intersection and overlap.

**Table 5:** The names of the eight clusters along with the primary keywords for each cluster.

#0	colorectal cancer	colorectal cancer; pancreatic cancer; immune response; bioinformatics tools; bacteria-fungi interaction; *Fusobacterium nucleatum*; tumor microbiome; bacteria; cancer; persistence
#1	inflammation	tumor microbiome; *Fusobacterium nucleatum*; bacteria; risk; cancer; pancreatic cancer; colorectal cancer; immune response; bioinformatics tools; bacteria-fungi interaction
#2	oral squamous cell carcinoma	oral squamous cell carcinoma; associated macrophage; hepatocellular carcinoma; preclinical mechanisms; anti-cancer therapy; tumor microenvironment; tumor microbiome; host-microbe interaction; cancer biology; oral bacteria
#3	immunotherapy	breast cancer; tumor microenvironment; intratumoral microbiome; immune cell; metabolic heterogeneity; immunotherapy; resistance; cancer; microbiome; efficacy
#4	porphyromonas gingivalis	colorectal cancer; tumorigenesis; molecular features; association; familial adenomatous polyposis; *Fusobacterium nucleatum*; pancreatic cancer; intratumoral microbiota; cervical cancer; poor prognosis
#5	hepatocellular carcinoma	hepatocellular carcinoma; metabolic pathway; fatty acid; lipid synthesis; bacterial theranostics; intratumoral microbiota; *Fusobacterium nucleatum*; pancreatic cancer; identification; osteomyelitis
#6	tumor microenvironment	tumor microenvironment; toll-like receptor; tumor microbiota; targeting therapy; host microbial interactions; intratumoral microbiota; tumor microenvironment; breast cancer; spatial multi-omics; targeting therapy
#7	intratumor bacteria	tumor microbiome; *Fusobacterium nucleatum*; tumor microenvironment; tumor-associated macrophages; colorectal cancer; intratumor bacteria; tumor metabolism; ellagic acid; targeted therapy; trastuzumab resistance
#8	tissue	cancer; *Fusobacterium nucleatum*; tumor microbiome; bacteria; persistence; gut microbiota; lung cancer; intratumoral microbiota; human papillomavirus; penile cancer
#9	immune microenvironment	immune microenvironment; therapeutic target; immune checkpoint inhibitor; mammary carcinoma; host microbial interactions; *Fusobacterium nucleatum*; cells; reveals; mutations; survival
#10	gut microbiota	gut microbiota; immune checkpoint inhibitors; immune-related adverse events; skin microbiota; tumor microbiome; gut microbiome; cancer immunotherapy; microbiome modulation; dietary interventions; candida albicans

The purpose of keyword burst analysis is to identify emerging concepts that have gained frequent citations during a specified period, thereby highlighting emerging research hotspots or frontiers within a particular field. This analysis enables researchers to anticipate future research directions and trends by monitoring the frequency and timing of keyword occurrences (**Figure 8**). The findings are based on a chronologically compiled and presented analysis of keyword appearances, with CiteSpace configured using the following parameters: γ (1.0), minimum duration = 1, as shown in **Figure 8**.

The keyword “*colon cancer*” is particularly prominent, exhibiting a burst strength of 4.64, reflecting significant attention between 2013 and 2019. Most keyword bursts are concentrated between 2016 and 2021. Notable early burst keywords include “*colon cancer*” (2013-2019) and “disease” (2014-2019). Both keywords began showing significant increases in citations in 2013 and 2014, respectively, and peaked in 2019. This trend reflects researchers’ early and sustained focus on the mechanistic progression of colon cancer. Furthermore, the keywords “*molecular features*” (2016-2022) and “*bacterial*” (2016-2022) have exhibited continuous bursts since 2016, indicating a deepening exploration of intratumoral microbiota as molecular marker features in tumors. The keyword “Kappa B” began attracting attention in 2023, reflecting a growing emphasis on the role of this key transcription factor in inflammatory and oncogenic processes. Meanwhile, the keyword “*binding*” shows a significant burst between 2024 and 2025, suggesting that exploring the close integration between microbiology and cancer biology, including research on immunotherapy targets, may represent an emerging frontier in current research.

The timeline view (**Figure 9**) illustrates the temporal evolution of research related to tumor-associated microbiota from 2012 to 2025, showing a gradual shift in research focus across different thematic clusters. From 2012 to 2015, research activity was primarily concentrated on gastrointestinal cancer topics. Keywords that emerged early in the timeline included “*cancer*”, “ *gut microbiome*”, “ *gastric cancer*”, “*colon cancer*”, “*diversity*”, “*colorectal cancer*”, “*bacteria*”, “*cells*”*, and* “*disease*”, indicating that early studies mainly focused on the association between intratumoral microbiota and gastrointestinal cancers. During this period, “*Fusobacterium nucleatum*” was a prominent keyword, reflecting its central role in research on microbiota associated with gastrointestinal tumors. Meanwhile, the early attention to “*colon cancer* “ and “*bacteria* “ demonstrated initial explorations into the role of microorganisms in the tumor microenvironment of colorectal cancer. In short, this stage was characterized by exploration and associative research themes.

Between 2015 and 2020, the timeline shows a shift toward more specific and complex interactions between microbiota and cancer processes. Keywords related to microorganisms that emerged included “*molecular features*”, “*Porphyromonas gingivalis*”, “*Salmonella typhimurium*”, “*Escherichia coli*”, “*tumor microbiome*”, “*oral microbiome*”, “*Bacteroides fragilis*”*, and* “*virus*”, while cancer-related keywords included “*cancer therapy*”, “*cancer progression*”, “*T cells*”, “ *tumor immunity*”, “*cancer progression*”, and “ *tumor microenvironment*”. This suggests that research from 2015 to 2020 was relatively focused on the interactions between specific bacterial species and cancer processes. Additionally, the appearance of keywords such as “*protein*, “ *microbial metabolites, and* “ *gene expression* indicated a growing interest in the mechanisms underlying tumor progression.

From 2020 to 2025, more keywords related to tumor-host interactions and tumor therapy frequently appeared on the timeline, including “ *identification*”, “*factor kappa B*”, “*clinical translation*”, “*chemoradiotherapy*”, “*persistence*”, “*immune microenvironment*”, “ *inhibition*”*, and* “ *risk factors*”. These keywords were primarily distributed within clusters related to tumor therapy themes, showing a trend toward integrating microbiota research with therapeutic strategies and immune responses, highlighting clinical translation.

### 3.6 Analysis of research trends and characteristics

To gain a comprehensive understanding of the developmental trends of tumor-associated microbiomes and their implications for cancer treatment, this study performed a summary analysis of preclinical and clinical trials regarding the application of intratumoral microorganisms in therapeutic interventions, as illustrated in **Table 6**, which includes a total of 69 studies labeled from *1 to *69. The abbreviations for tumor sites in the table are as follows: Bca (breast cancer), NPC (nasopharyngeal carcinoma), Lc (lung cancer), CRC (colorectal cancer), ESCA (esophageal carcinoma), PDAC (pancreatic cancer), MEL (melanoma), and PCa (prostate cancer), CC (Cervical cancer), OSCC (oral squamous cell carcinoma), SCC (squamous cell carcinoma). The studies are primarily categorized into five types: Animal testing (62 studies), Nano Research (19 studies), Engineered bacteria (3 studies), RCT (2 studies), Phase I Clinical Trial (1 studies) and *In vitro* experiments (2 studies). Given that both Nano Research and Engineered bacteria involve Animal testing, the total number of studies exceeds 69. Post-intervention primary outcomes include Endocrine and targeted therapy (11 studies), Chemotherapy (20 studies), Intratumoral bacterial clearance (17 studies), and Immunotherapy (21 studies). When considered in conjunction with the results in the table, we find that tumor immunotherapy constitutes a substantial proportion of the studies, which is consistent with the results of the keyword clustering. The symbols “+” and “-” denote whether the reported effects of intratumoral bacteria on tumor treatment were positive or negative. Analysis reveals that most studies reported negative outcomes (21 studies with “+” and 48 studies with “-”), indicating that the presence of intratumoral bacteria is often detrimental to the tumor treatment process, which necessitates interventions targeting these bacteria to enhance anti-tumor therapies. Notably, research on *Fusobacterium nucleatum* is the most prevalent, with 25 studies, which aligns with the co-occurrence and clustering results of keywords, thereby suggesting that *Fusobacterium nucleatum* is currently a focal point in the study of intratumoral microbiota.

**Table 6:** Current preclinical research and clinical trials of intratumoral microorganisms in tumor treatment interventions.

Endocrine and targeted therapy					
Sequence Number	Year	Tumor site	Classification of interventional studies	Types of microorganisms	Effect
*1 (30)	2025	Bca	Animal testing	*Firmicutes Lactobacillus*	+
*2 (31)	2023	CRC and Bca	Animal testing	*Proteus mirabilis* (A‐gyo) and *Rhodopseudomonas palustris* (UN‐gyo)	+
*3 (32)	2017	RCC, CRC, READ	Animal testing	non-pathogenic *E. coli* strain MG1655	+
*4 (33)	2025	Bca	Animal testing	*Pseudomonas aeruginosa* 3oc	–
*5 (34)	2024	NPC	Animal testing	*Lactobacillus plantarum*	+
*6 (35)	2024	Bca	Animal testing	Tumor-colonizing microorganisms	–
*7 (36)	2024		Animal testing	Tumor-colonizing microorganisms	+
*8 (37)	2023	Lc	Animal testing	*Akkermansia muciniphila*	+
*9 (38)	2022	CRC, Lc and Bca	Nano Research, Animal testing	Intratumoral Microbiome	+
*10 (39)	2022	Bca	Animal testing	*Helicobacter hepaticus*	–
*11 (40)	2020	OSCC	Animal testing	*Porphyromonas gingivalis, Treponema denticola, and Fusobacterium nucleatum*	–
Chemotherapy					
*12 (41)	2024	CRC	Nano Research, Animal testing	*Fusobacterium nucleatum*	–
*13 (42)	2024	Bca	Nano Research, Animal testing	*Fusobacterium nucleatum*	–
*14 (43)	2023	CRC	Nano Research, Animal testing	*Fusobacterium nucleatum*	–
*15 (44)	2023	CRC	Nano Research, Animal testing	Intratumoral Microbiome	–
*16 (45)	2025	Bca	Nano Research, Animal testing	Intratumoral Microbiome	–
*17 (46)	2024	CRC	Nano Research, Animal testing	*Fusobacterium nucleatum*	–
*18 (47)	2022	PDAC	Nano Research, Animal testing	*Fusobacterium nucleatum*	–
*19 (48)	2023	PDAC, Bca	Engineered bacteria, Animal testing	HAase-expressing *Salmonella*	+
*20 (49)	2022	CRC	Animal testing	*Fusobacterium nucleatum*	–
*21 (50)	2023	CRC	Nano Research, Animal testing	*Fusobacterium nucleatum*	–
*22 (51)	2022	ESCA	Animal testing	*Fusobacterium nucleatum*	–
*23 (52)	2025	CRC	Nano Research, Animal testing	*Fusobacterium nucleatum*	–
*24 (53)	2024		Animal testing	*Pseudomonas aeruginosa*	–
*25 (54)	2024	CRC	Nano Research, Animal testing	*Fusobacterium nucleatum*	–
*26 (55)	2024		Animal testing	*Pseudomonas aeruginosa*	–
*27 (56)	2019		Animal testing	Intratumoral Microbiome	–
*28 (45)	2025	Bca	Nano Research, Animal testing	Intratumoral Microbiome	+
*29 (57)	2023	CC	Animal testing	*Lactobacillus iners*	–
*30 (58)	2017		Animal testing	Bacteria containing the long CDD variant	–
*31 (59)	2023	Lc	*In vitro* experiments	*Staphylococcus aureus*	–
Intratumoral bacterial clearance					
*32 (60)	2021	CRC	RCT	*Fusobacterium nucleatum*	–
*33 (61)	2025	CRC	Animal testing	Intratumoral Microbiome	–
*34 (44)	2023		Nano Research, Animal testing	Intratumoral Microbiome	–
*35 (26)	2024	Bca	Nano Research, Animal testing	*Fusobacterium nucleatum*	–
*36 (62)	2025		Animal testing	*Fusobacterium nucleatum*, *Streptococcus sanguinis*, *Enterococcus faecalis*, and *Staphylococcus xylosus*	–
*37 (63)	2022		Engineered bacteria, Animal testing	Intratumoral Microbiome	–
*38 (64)	2025		Nano Research, Animal testing	Intratumoral Microbiome	–
*39 (65)	2017		RCT	*Fusobacterium nucleatum*	–
*40 (66)	2023	CRC	Nano Research, Animal testing	*Fusobacterium nucleatum*	–
*41 (67)	2021	Bca	RCT	Intratumoral Microbiome	–
*42 (68)	2025		Animal testing	Intratumoral Microbiome	–
*43 (69)	2024	PDAC	Animal testing	*Fusobacterium nucleatum*	–
*44 (54)	2024	CRC	Animal testing	*Fusobacterium nucleatum*	–
*45 (70)	2022	Bca	Animal testing	*Streptococcus*, *Lactobacillus*, *Staphylococcus*, and *Enterococcus*	–
*46 (71)	2025		Animal testing	*Escherichia coli*	–
*47 (72)	2025	Bca	Nano Research	*Fusobacterium nucleatum*	–
*48 (73)	2023	CRC	Animal testing	*Fusobacterium nucleatum*	–
Immunotherapy					
*49 (74)	2022	PDAC	Animal testing	Fungus	–
*50 (75)	2023	MEL	Animal testing	*Lactobacillus reuteri*	+
*51 (76)	2021		Phase I Clinical Trial	mycobacteria	+
*52 (77)	2024	CRC	RCT, Animal testing	*Escherichia coli* Nissle 1917	+
*53 (78)	2024	PDAC	Animal testing	*Proteobacteria*	+
*54 (79)	2024	CRC	Animal testing	Intratumoral Microbiome	+
*55 (80)	2023	ESCA	Animal testing	*Fusobacterium nucleatum*	–
*56 (27)	2023	PDAC	Nano Research, Animal testing	*Fusobacterium nucleatum*	–
*57 (81)	2025		Animal testing	*B. parabrevis*	+
*58 (82)	2020		Animal testing	*Bifidobacteria*	+
*59 (83)	2024	CRC	Animal testing	*Fusobacterium nucleatum*	+
*60 (84)	2023	ESCA	Animal testing	*Streptococcus*	+
*61 (85)	2022	SCC	*In vitro* experiments	*Fusobacterium*	–
*62 (86)	2024	Bca	Nano Research, Animal testing	Intratumoral Microbiome	+
*63 (87)	2025	Bca	Animal testing	*Stenotrophomonas maltophilia*	+
*64 (88)	2022	CRC	Engineered bacteria, Animal testing	*Fusobacterium nucleatum*, *Escherichia coli*, *Staphylococcus aureus*, *Bifidobacterium pseudolongum*	+
*65 (89)	2018	PDAC	Animal testing	Intratumoral Microbiome	–
*66 (90)	2022	OSCC	Animal testing	*Peptostreptococcus*	–
*67 (91)	2021		Nano Research, Animal testing	Intratumoral Microbiome	–
*68 (74)	2022		Animal testing	Fungus	–
*69 (92)	2025	Bca	Animal testing	*Fusobacterium nucleatum*	–

## 4 Discussion

This study, based on bibliometric analysis, systematically mapped the knowledge structure and evolutionary trajectory of research on intratumoral microbiota, and examined its potential value in tumor initiation, progression, and clinical applications. The results show that research on intratumoral microbiota began to emerge in 2012 and entered a phase of rapid development after 2020. This is in agreement with the results reported by Xiao et al (93). This shift indicates that intratumoral microbiota is gradually becoming a frontier field at the intersection of oncology and immunology. Next, we will discuss the results of the search and analysis conducted using Web of Science and PubMed.

### 4.1 Evolution and hotspots of research themes

According to our timeline results, research on the intratumoral microbiome has evolved from the characterization of cancer-associated microbial communities and extensive studies in colorectal cancer to the investigation of specific microbial species and their mechanisms, ultimately progressing to immune modulation and therapeutic interventions. Among them, the themes related to intratumoral microorganisms, immunotherapy, and the immune microenvironment have gradually assumed significant prominence in recent years, as evidenced by keyword co-occurrence and temporal evolution analyses. Early studies primarily utilized 16S rRNA sequencing to observe microbial distribution, revealing associations between microbiota such as *Helicobacter pylori* and *Fusobacterium nucleatum* with gastric cancer and colorectal cancer (94, 95). The advent of multi-omics technologies (96) and later developments in spatial transcriptomics have enabled researchers to trace the spatial co-localization relationships between microbiota and host cells at both single-cell and subcellular scales. Furthermore, this reveals the intricate interactions between complex microbial communities and the cancer process. In recent years, the integration of immunotherapy, outer membrane vesicle (OMV) coated nanotechnology (27), electronic antibacterial therapy (EAT) (69), and nanocarrier technology (44) has further emphasized the potential value of tumor-associated microorganisms as therapeutic targets and supportive factors. In summary, this evolutionary trend indicates that research on tumor-associated microorganisms is progressively transitioning from correlation validation to functional mechanism elucidation and ultimately to the exploration of clinical applications.

### 4.2 The application of tumor-associated microorganisms in cancer therapy

Through keyword clustering analysis, we found that themes related to tumor immunotherapy exhibit a high degree of clustering in the research on intratumoral microorganisms, suggesting that this field is gradually becoming one of the important application directions of current research. The results of the keyword co-occurrence analysis indicate that “*immunity*” has a count of 117 and a centrality of 0.02. Furthermore, the results of the intervention subset reveal that “*cancer immunotherapy*” is one of the therapeutic approaches with significant clinical translational trends, which accounts for a considerable proportion of the studies on intratumoral microorganisms and tumor treatment. Based on our findings, three key points regarding cancer treatment are worthy of discussion, including functional heterogeneity, diversity of intervention strategies, and the synergistic regulation of representative target bacteria and overall microecology. Firstly, tumor-associated microbiota may exert dual effects—either promoting or inhibiting cancer treatment. As shown in **Table 5**, different microbiota demonstrates certain variations in immunotherapy, endocrine therapy, targeted therapy, and chemotherapy. On the one hand, the studies summarized in the table indicate that certain naturally occurring intratumoral microorganisms or tumor-associated commensals, including *Lactobacillus*, *Akkermansia muciniphila*, and *Bifidobacterium*, are associated with enhanced CD8^+^ T-cell infiltration, induction of immunogenic cell death, and modulation of the tumor immune microenvironment, thereby potentiating the efficacy of immune checkpoint inhibitors, chemotherapy, or targeted therapies. On the other hand, several studies employed engineered or exogenously administered bacteria (e.g., *Salmonella* or *Escherichia coli* Nissle 1917) as therapeutic tools. Although these bacteria are not intrinsic components of the intratumoral microbiota, they can exert antitumor effects by selectively eliminating deleterious intratumoral microorganisms, reshaping the tumor microenvironment, and/or directly enhancing antitumor immune responses. Together, this evidence suggests that tumor-associated microbiota constitutes an important regulatory factor influencing responses to cancer treatment, rather than simply being classified as “harmful” or “beneficial”. Furthermore, unlike the non-specific bactericidal effects of traditional antibiotics, recent years have seen a substantial focus on the precise modulation of intra-tumoral microbiota rather than their complete eradication. As shown in **Table 5**, emerging strategies such as nanodrug delivery systems, biomimetic nanomaterials, outer membrane vesicle coatings, photothermal/photodynamic therapy, and electroporation antibacterial treatments have been widely used to target tumor-associated microbiota. These methods have achieved the goal of “specifically eliminating pathogenic bacteria while preserving or reshaping beneficial microbiota” in various tumor models, and they can synergize with chemotherapy, immunotherapy, or targeted therapy. Additionally, engineered bacteria can serve not only as antibacterial tools but also as drug delivery carriers or immune activation platforms, further expanding the therapeutic dimensions of tumor microbiota intervention. However, most current studies remain at the animal experiment or early clinical stages, and their long-term safety, stability of microbiota remodeling, and optimal administration routes still require further validation. Moreover, as indicated by the distribution of interventional studies included in the table, current research often focuses on several representative microorganisms in practical applications. Among them, *Fusobacterium nucleatum* has become one of the most used model target bacteria in nanodrug, antibacterial vaccine, and combination therapy studies due to its stable colonization in various gastrointestinal tumors and breast cancer, its relatively clear pathogenic mechanisms, and the presence of defined molecular targets. However, the table also shows that *Escherichia coli*, *Pseudomonas aeruginosa*, *Streptococcus*, *Proteobacteria*, and intra-tumoral fungal communities play important roles in regulating treatment sensitivity and the immune microenvironment. An increasing number of studies are beginning to focus on the combined effects of multi-species coexistence and their interactions within tumors on treatment responses, attempting to achieve systematic improvement in therapeutic outcomes by eliminating key pathogenic bacteria, modulating functional microbiota modules, or overall remodeling of the tumor microecological network. Overall, existing evidence indicates that tumor-associated microbiota has become a key regulatory factor influencing cancer treatment responses and prognosis. Future research needs to advance from correlational descriptions to elucidating mechanisms and establishing causal relationships, and through multi-omics integration and clinical translational studies, explore safer, more precise, and scalable tumor microbiota intervention strategies, thereby providing a new theoretical basis and therapeutic targets for personalized cancer treatment.

### 4.3 Tumor colonization by gut microbiota

During the literature screening, keyword co-occurrence analysis, and clustering analysis, we observed that “gut microbiota” emerged as a high-frequency keyword and simultaneously exhibited a significant betweenness centrality (0.25) in the study of intratumoral microbiota, indicating that the gut microbiome has become an important and indispensable topic of association in this research field. Upon reviewing relevant literature for further analysis, we discovered that current research involving gut microbiota and intra-tumoral microbiota predominantly focuses on describing key microbial populations that have been repeatedly reported in recent gut microbiome studies, which are also detected in the tumor microenvironment, such as *Lactobacillus*, *Clostridium, Bifidobacterium*, and *Akkermansia muciniphila* (37, 82, 97, 98). These studies, to some extent, expand our understanding of the sources and distribution of tumor-associated microbiota; however, they primarily remain phenomenological in nature. In recent years, numerous studies have begun to investigate the potential functions of these microorganisms within the microenvironment. Yet, systematic research supporting the overall changes and interaction patterns between the gut microbiome and the intra-tumoral microbiome is still lacking. This is particularly evident in the intervention aspect, as the number of studies directly assessing the impact of rapidly developing fecal microbiota transplantation (FMT) methods from gut microbiome research on intra-tumoral microbiota remains limited. This phenomenon reflects a significant methodological bottleneck faced by the field, specifically concerning whether and under what conditions gut-derived microorganisms can effectively colonize and persist stably at tumor sites. Notably, recent studies on “gut microbiota colonization of tumors” have provided new perspectives on this issue. Some research has observed the accumulation of gut-derived microorganisms within tumor tissues (82, 99). Additionally, mechanisms linking host molecular characteristics with the enrichment of specific microorganisms within tumors have been reported. Shi et al. found that KRAS mutations can actively promote the intratumoral colonization of toxin-producing *Bacteroides fragilis* in colorectal cancer by modulating the miRNA3655/SURF6/IRF7/IFNβ axis (references) (100). This shift indicates that research interest is transitioning from “whether intra-tumoral microbiota exist” to “which factors determine their colonization and stability.” Based on existing literature and keyword co-occurrence analysis results, this study posits that research surrounding the relationship between gut microbiota, fecal microbiota transplantation, and intra-tumoral colonization is forming a new interdisciplinary hotspot. This also leads to a noteworthy research direction: whether it is possible to enhance the colonization potential of beneficial microorganisms at tumor sites by modulating host-related molecular characteristics or microenvironment conditions, thereby providing new strategies for the precise regulation of the tumor microenvironment. The increasing attention to this direction suggests its potential significance in future tumor microbiota research and adjunct strategies for immunotherapy.

### 4.4 Tumor-associated microorganisms and their metabolites in relation to tumor immunity

The sustained burst of the keyword “molecular features” (2016-2022) indicates that research on the intratumoral microbiota has entered a phase of deep mechanistic exploration during this period. Combined with the results from the timeline chart, both tumor-associated microorganisms themselves and their metabolites, serving as molecular features, are currently one of the hot topics in the field of tumor-associated microbiota research. While the keyword “binding” is projected to show a significant burst between 2024 and 2025, its meaning is relatively broad, encompassing areas such as the integration of multi-omics data and the binding of therapeutic targets to drugs. Based on this, we attempt to discuss the feasibility of combining metabolites with immunotherapy as a future research direction. Short-chain fatty acids, particularly butyrate, have been repeatedly reported as key immunomodulatory metabolites (101–103). For example, butyrate derived from tumor-associated can enhance the T cell receptor (TCR) signaling of cytotoxic CD8^+^ T cells, thereby improving their effector function and enhancing the anti-tumor efficacy of immune checkpoint inhibitors such as anti-PD-1 (104). Such studies provide a more mechanistic explanation for the association between “microbiota—immunotherapy response” from the perspective of immunometabolism, suggesting that metabolites may serve as critical mediating factors in regulating sensitivity to immunotherapy. In contrast to immune-enhancing metabolites, certain microbially-associated metabolic products have been confirmed to participate in shaping an immunosuppressive tumor microenvironment. The colonization and metabolic activity of symbiotic microorganisms, particularly anaerobes, in hypoxic tumor environments can also significantly promote the local accumulation of lactate, thereby exacerbating tumor microenvironment acidification. This lactate-enriched microenvironment can weaken anti-tumor immune responses through various pathways, promoting tumor progression and immune evasion. Recent studies have shown that elevated lactate levels in the tumor microenvironment can downregulate the expression of PPARγ in invariant natural killer T (iNKT) cells, thereby inhibiting cholesterol biosynthesis and IFN-γ production, which weakens their anti-tumor immune effects (105). Additionally, delivery systems targeting the metabolism of symbiotic anaerobic bacteria under hypoxic conditions can reduce lactate accumulation within tumors, reversing lactate-mediated immunosuppression and chemotherapy resistance (106). These findings suggest that microbially-mediated metabolic alterations may serve as significant functional hubs linking immunosuppression, tumor progression, and therapeutic resistance. In addition to single metabolomics studies, an increasing number of multi-omics integration analyses have further revealed the systematic associations among tumor-associated microorganisms, metabolic remodeling, and immune heterogeneity (10, 96, 107–109). This research trend emphasizes that metabolic changes are not isolated events, but rather key regulatory layers embedded within the tumor immune ecosystem. Although most current studies still focus on correlation analysis, the introduction of metabolomics has significantly enhanced the depth of immunological interpretations, providing new theoretical support for understanding how microorganisms regulate anti-tumor immunity. In the future, systematic research combining metabolomics, immune phenotype analysis, and functional experiments will contribute to further clarifying the causal role of microbially-mediated metabolic changes in tumor immune regulation, advancing its clinical translation toward predictive immunotherapy and combined intervention strategies.

### 4.5 Advances and challenges in detection technologies

The extremely low abundance of intratumoral microorganisms has led to ongoing debates regarding detection methods. In recent years, emerging technologies such as simplified metagenomic sequencing (2bRAD-M), upgraded 16S sequencing, spatial transcriptomics, and SLACS have significantly enhanced the ability to analyze low-abundance, spatial heterogeneity, and functional distribution (21, 110, 111). Additionally, the application of artificial intelligence and big data algorithms has made it possible to identify intratumoral microorganisms across different cancer types (20). However, challenges such as contamination control, sample standardization, and data consistency remain pressing issues in this field. A research team from Johns Hopkins University (112) reanalyzed whole-genome sequencing (WGS) data of 5,734 samples covering 25 cancer types from The Cancer Genome Atlas (TCGA) project, revealing that the presence of microorganisms in tumors is far less than previously reported, with sequencing read errors potentially reaching tens of thousands. This has cast even greater doubt on the actual existence and abundance assessment of intratumoral microorganisms. Effectively distinguishing genuine low-biomass microbial colonization from exogenous or environmental contaminants introduced during experimental procedures has become a core methodological challenge in the field of intratumoral microbiology. Without stringent quality control, microbial signals can easily be obscured by exogenous contamination. Therefore, the core principle guiding experimentation is that contamination control must be treated as a paramount consideration at every step—from experimental design and sample processing to data analysis—otherwise, microbial signals are highly susceptible to being masked by contamination. Additionally, there is an urgent need to establish unified detection and analytical protocols across different centers. This includes standardized protocols for sample collection and preservation, the standardized use of core reagents, and consistent bioinformatic analysis workflows and parameters.

### 4.6 Future perspectives

Future research directions can be deepened from the following aspects: 1. The first and most important point is: The lack of consistency across studies is particularly problematic in the context of intratumoral microbiomes, where microbial abundance is extremely low and sequencing artifacts can dominate the signal. Inadequate contamination control and heterogeneous analytical pipelines may lead to overestimation or misclassification of microbial presence, underscoring the urgent need for standardized methodologies and rigorously curated reference datasets. 2. Mechanistic studies should move beyond descriptive associations to clarify how intratumoral microorganisms functionally interact with distinct cellular components of the tumor microenvironment. In particular, the causal relationships between intratumoral microbes, immune cell subsets, cancer-associated fibroblasts, and local metabolic alterations remain poorly defined. Elucidating how these interactions evolve during tumor progression and treatment will be essential for distinguishing passenger microbes from functionally relevant regulators. 3. Greater emphasis should be placed on multi-omics approaches. While single-omics studies have provided important insights, they are often insufficient to capture the spatial heterogeneity of intratumoral microorganisms and their molecular effects. Combining spatial transcriptomics, metabolomics, and microbiome profiling at the tissue level may enable more accurate identification of microbe-associated molecular and immune phenotypes that are clinically relevant. 4. Current research remains heavily bacteria-centered, which likely underestimates the complexity of the intratumoral microecosystem. Emerging evidence suggests that fungi, viruses, and bacteriophages, as well as their interactions with bacteria, may influence tumor biology and immune regulation. Expanding research beyond bacteria alone will be necessary to construct a more complete and biologically meaningful map of the intratumoral microbiome. 5. Clinical translation and novel interventions: “Microbiota-targeted therapy” is gradually emerging as a potential clinical intervention strategy, which involves selectively modulating tumor-associated microorganisms and their functional states to improve the tumor immune microenvironment and therapeutic responses. For instance, the precise elimination of tumor-promoting or immunosuppressive bacterial strains using agents such as sterilizing vaccines and nanocarriers can alleviate immune suppression and reduce the risk of resistance; engineered bacteria can be utilized for localized delivery to produce relevant metabolites that modulate the immune microenvironment, thereby enhancing the efficacy of immunotherapy. But their efficacy, controllability, and long-term safety require further clinical validation.

Lastly, this study also has certain limitations. First, bibliometric analysis relies on databases and search strategies, which may result in the omission of some non-indexed or non-English literature. Second, bibliometric indicators themselves cannot directly reflect research quality or clinical value and should be supplemented by systematic reviews and mechanistic experiments. Additionally, given that research on intratumoral microbiomes is still rapidly evolving, the analysis of trends and hotspots may shift over time; therefore, conclusions need to be updated dynamically. In addition, when analyzing the status and trends of preclinical and clinical trial research on intratumoral microbiomes, several limitations should be acknowledged. First, database searches can be broadened to capture a more comprehensive set of sources. Second, the field is rapidly evolving, so trends identified at one time may shift with new technologies and approaches, necessitating periodic updates. Third, the methodological quality of included studies varies, introducing risk of bias in summaries and conclusions.

## 5 Conclusion

This study analyzed the current research status of intratumoral microbiomes based on bibliometric methods. Current literature identifies several stable hotspot areas. Immuno-oncology remains the core field, with intratumoral microorganisms implicated in the modulation of anti-tumor immunity, and their effects on responses to immune checkpoint inhibitors and the tumor microenvironment vary across different cancer contexts. Advances in intratumoral microbial mechanisms are driven by multi-omics integration, with particular emphasis on the connections between microbial metabolites, host immunometabolism pathways, and the tumor microenvironment. The gut–tumor axis and colonization by gut-derived microbes have become particularly active research directions, spurring investigations into determinants of colonization and potential regulatory strategies to support therapy. Therapeutic strategies, including nano-delivery systems, probiotics, and engineered bacteria—are increasingly represented, but most remain at the preclinical stage, with unresolved questions regarding safety, durability, and translational feasibility. Diagnostic and prognostic biomarker research is evident but remains exploratory, requiring validation across cohorts. Collectively, these hotspots suggest the field is moving toward mechanistic understanding and translational exploration, while emphasizing the need for robust validation and cross-institutional collaboration to translate findings into safe and effective clinical interventions.

## Data Availability

The original contributions presented in the study are included in the article/**Supplementary Material**. Further inquiries can be directed to the corresponding author.
