# Supplementary material for: Research on microbial communities in tumor microenvironments: cutting-edge dynamics and future trends from a bibliometric perspective
Source: Front Immunol. 2026 Feb 16;17:1745842. doi: 10.3389/fimmu.2026.1745842 (PMC12950562; doi:10.3389/fimmu.2026.1745842)
Supplement: Supplementary file 1 [file DataSheet1.docx]

Web of Science:

# Searches:

1: TS =("tumor microb*" OR "tumour microb*" OR "tumoral microb*" OR "tumoural microb*" OR "tumor mycob*" OR "tumour mycob*" OR "tumoral mycob*" OR"tumoural mycob*" OR "tumor bacter*" OR "tumour bacter*" OR "tumoral bacter*"OR"tumoural bacter* " OR "tumor fung*" OR "tumour fung*" OR "tumoral fung*"OR"tumoural fung*" OR "intratumor microb*" OR "intratumour microb*" OR "intratumoral microb*" OR "intratumoural microb*" OR "intratumor mycob*" OR"intratumour mycob*" OR "intratumoral mycob*" OR "intratumoural mycob*" OR"intratumor bacter*" OR "intratumour bacter*" OR "intratumoral bacter*" OR"intratumoural bacter*" OR "intratumor fung*" OR "intratumour fung*" OR"intratumoral fung*" OR "intratumoural fung*" OR "tumor-resident microb*" OR "tumour resident microb*" OR "tumor-resident bacter*" OR "tumour-resident bacter*" OR "tumor-resident mycob*" OR "tumour-resident mycob*" OR"tumor-resident fung*" OR "tumour-resident fung*" OR "tumor-associated microb*"OR"tumour-associated microb*" OR "tumor-associated bacter*" OR "tumour-associated bacter*" OR "tumor-associated mycob*" OR "tumour-associated mycob*" OR "tumor-associated fung*" OR "tumour-associated fung*" OR "tumor-related microb*" OR "tumour-related microb*" OR "tumor-related bacter*" OR "tumour-related bacter*" OR "tumor-related mycob*" OR "tumour-related mycob*" OR "tumor-related fung*" OR "tumour-related fung*" OR "tumor tissue microbiome" OR "in-tumor microb*") Date Run: Mon Dec 22 2025 Results: 1289

2: intratumoral microbiome (All Fields) OR intratumoral microbiota (All Fields) OR in-tumor microbiome (All Fields) OR tumor-resident microbiome (All Fields) OR tumor tissue microbiome (All Fields) Date Run: Mon Dec 22 2025 Date Run: Mon Dec 22 2025 Results: 2222

3: #1 OR #2 Results: 2835

4: #1 OR #2 and Letter or Proceeding Paper or News Item or Book Chapters or Correction or Retracted Publication or Expression Of Concern or Data Paper or Publication With Expression Of Concern or Retraction or Meeting Abstract or Editorial Material or Early Access (Exclude – Document Types) Results: 2512

PubMed

(("Microbiota"[MeSH Terms] OR "Bacteria"[MeSH Terms] OR "Fungi"[MeSH Terms] OR microbiome[Title/Abstract] OR microbiota[Title/Abstract] OR bacterium[Title/Abstract] OR bacteria[Title/Abstract] OR fungal[Title/Abstract] OR fungi[Title/Abstract] OR microbe[Title/Abstract])) AND (( "tumor resident"[Title/Abstract] OR "tumour resident"[Title/Abstract] OR "tumor tissue"[Title/Abstract] OR "tumour tissue"[Title/Abstract] OR intratumoral[Title/Abstract] OR "tumor tissues" [Title/Abstract] OR intratumoural[Title/Abstract] OR intratumor[Title/Abstract] OR intratumour[Title/Abstract] OR in tumor[Title/Abstract])) OR (("bacterial colonization"[Title/Abstract]) AND (cancer[Title/Abstract] OR tumor[Title/Abstract])) OR ("tumor-colonized bacteria"[Title/Abstract])

Filters: Clinical Study, Clinical Trial, Clinical Trial, Phase I, Clinical Trial, Phase II, Clinical Trial, Phase III, Clinical Trial, Phase IV, Controlled Clinical Trial, Pragmatic Clinical Trial, Randomized Controlled Trial, Research Support, N.I.H., Extramural, Research Support, N.I.H., Intramural, Research Support, Non-U.S. Gov't, Research Support, U.S. Gov't, Non-P.H.S., Research Support, U.S. Gov't, P.H.S., Research Support, U.S. Gov't

Date Run: Mon Dec 22 2025

Results: 1867

Time Span: From the establishment of the database to December 22, 2025

Language Filter: No language filter applied

Export Format: CSV format

Record Saving: Full records

Seach in: Web of Science Core Collection

Collections: Web of Science Core Collection and Chinese Science Citation Database^sm^
