# Supplementary material for: Research on microbial communities in tumor microenvironments: cutting-edge dynamics and future trends from a bibliometric perspective
Source: Front Immunol. 2026 Feb 16;17:1745842. doi: 10.3389/fimmu.2026.1745842 (PMC12950562; doi:10.3389/fimmu.2026.1745842)
Supplement: Supplementary file 2 [file DataSheet2.docx]

# Edit this file in Excel or Notepad++ and save it as citespace.alias

# Modify the first column as the label to retain

"# Remove lines that don't need changes, or simply leave them alone"

# Entries are arranged from the most cited to the least cited. You may only need to modify the first few lines

cancer cancer

tumor microbiome tumor microbiome

colorectal cancer colorectal cancer

fusobacterium nucleatum fusobacterium nucleatum

gut microbiome gut microbiota

bacteria bacteria

cells cells

tumor microbiome microbiome

tumor microenvironment tumor microenvironment

expression expression

gut microbiome gut microbiome

inflammation inflammation

risk risk

pancreatic cancer pancreatic cancer

association association

therapy resistance resistance

breast cancer breast cancer

cancer therapy therapy

immunotherapy immunotherapy

tissue tissue

ntratumor microbiome intratumoral microbiota

carcinogenesis carcinogenesis

lung cancer lung cancer

gut microbiome intestinal microbiota

tumor immunity immunity

activation activation

cancer tumor

helicobacter pylori helicobacter pylori

therapeutic efficacy efficacy

diversity diversity

health health

survival survival

intratumor microbiome intratumoral microbiome

t cells t cells

tumorigenesis tumorigenesis

chemotherapy chemotherapy

gastric cancer gastric cancer

infection infection

receptor receptor

gene gene

hepatocellular carcinoma hepatocellular carcinoma

cancer progression progression

disease disease

dysbiosis dysbiosis

mechanisms mechanisms

induction induction

chain fatty acids chain fatty acids

growth growth

pancreatic cancer pancreatic ductal adenocarcinoma

microbiota microbiota

colon cancer colon cancer

oral microbiota oral microbiota

porphyromonas gingivalis porphyromonas gingivalis

tumor immunity antitumor immunity

identification identification

gut gut

microbial metabolites metabolites

squamous cell carcinoma squamous cell carcinoma

protein protein

sequences sequences

intratumor bacteria intratumoral bacteria

periodontal disease periodontal disease

human papillomavirus human papillomavirus

cancer therapy cancer therapy

microbial metabolites metabolism

helicobacter pylori helicobacter pylori infection

probiotics probiotics

16s rna sequencing 16s rrna

tumor metastasis metastasis

bacterial bacterial

immune microenvironment tumor immune microenvironment

colonization colonization

molecular features molecular features

binding binding

proliferation proliferation

oral microbiome oral microbiome

therapeutic efficacy responses

carcinoma carcinoma

16s rna sequencing 16s rrna sequencing

delivery delivery

head head

model model

escherichia coli escherichia coli

gene expression gene expression

fusobacterium nucleatum <italic>fusobacterium nucleatum</italic>

gemcitabine gemcitabine

epidemiology epidemiology

16s rna sequencing 16s rrna gene

microenvironment microenvironment

prostate cancer prostate cancer

impact impact

dna dna

antibiotics antibiotics

intratumor microbiome intratumor microbiota

intratumor microbiome intratumor microbiome

oral squamous cell carcinoma oral squamous cell carcinoma

adenocarcinoma adenocarcinoma

double strand breaks double strand breaks

macrophages macrophages

biomarkers biomarkers

t cells regulatory t cells

immune microenvironment immune microenvironment

inhibition inhibition

tumor immunity innate

butyrate butyrate

cervical cancer cervical cancer

persistence persistence

aryl hydrocarbon receptor aryl hydrocarbon receptor

apoptosis apoptosis

intratumor bacteria intratumor bacteria

human microbiome human microbiome

immunotherapy cancer immunotherapy

alignment alignment

gut microbiome human gut microbiome

hypoxia hypoxia

hallmarks hallmarks

immunotherapy blockade

bladder cancer bladder cancer

tumor microenvironment tumour microenvironment

lung cancer lung adenocarcinoma

kappa b kappa b

cancer progression cancer progression

potential role potential role

pathway pathway

lung microbiome lung microbiome

open label open label

cells cells

promotes promotes

esophageal cancer esophageal cancer

salmonella typhimurium salmonella typhimurium

immunotherapy ctla 4 blockade

intracellular bacteria intracellular bacteria

in vivo in vivo

fusobacterium nucleatum fap2

tumor microbiome tumor microbiota

landscape landscape

liver cancer liver cancer

barrier barrier

bacteroides fragilis bacteroides fragilis

colon colon

acid acid

bile acids bile acids

immunotherapy immune checkpoint inhibitors

genome genome

virus virus

drug delivery drug delivery

mycobiome mycobiome

commensal bacteria commensal bacteria

infections infections

16s rna sequencing 16s rrna gene sequencing

machine learning machine learning

fecal microbiota transplantation fecal microbiota transplantation

reveals reveals

liver liver

suppressor cells suppressor cells

colorectal carcinogenesis colorectal carcinogenesis

epithelial cells epithelial cells

microbial metabolites microbial metabolites

ovarian cancer ovarian cancer

tumor metastasis tumor metastasis

cancers tumors

liver metastasis liver metastasis

epithelial-mesenchymal transition epithelial-mesenchymal transition

nanoparticles nanoparticles

cancer risk cancer risk

nasopharyngeal carcinoma nasopharyngeal carcinoma

cancer progression tumor progression

oral cancer oral cancer

outer membrane vesicles outer membrane vesicles

risk factors risk factors

immunotherapy nivolumab

microbial biomarker microbial biomarker

sp nov sp nov

obesity obesity

recurrence recurrence

tumor immunity immune response

mouse model mouse model

staphylococcus aureus staphylococcus aureus

melanoma melanoma

community community

dna damage dna damage

beta catenin beta catenin

cancer treatment cancer treatment

hepatitis b virus hepatitis b virus

attenuated salmonella typhimurium attenuated salmonella typhimurium

cell death cell death

hepatitis b hepatitis b

candida albicans candida albicans

breast tissue breast

cells cancer cells

therapy resistance chemoresistance

database database

engineered bacteria engineered bacteria

potentiates intestinal tumorigenesis potentiates intestinal tumorigenesis

growth factor growth factor

fusobacterium nucleatum nucleatum

breast tissue breast tissue

dendritic cells dendritic cells

commensal fungi commensal fungi

antibacterial antibacterial

head and neck cancer head and neck cancer

fusobacterium nucleatum fusobacterium

bacillus calmette guerin bacillus calmette guerin

chemoradiotherapy chemoradiotherapy

ductal adenocarcinoma ductal adenocarcinoma

therapy resistance drug resistance

features features

gut microbiome fecal microbiota

barretts esophagus barretts esophagus

cutibacterium acnes cutibacterium acnes

esophageal cancer esophageal squamous cell carcinoma

tumor immunity immune system

ulcerative colitis ulcerative colitis

deoxycholic acid deoxycholic acid

commensal commensal

anticancer anticancer

invasion invasion

bacterial infection bacterial infection

mucosal microbiota mucosal microbiota

kegg modules kegg modules

16s rna sequencing 16s rna sequencing

t cells cd8(+) t cells

dietary fiber dietary fiber

tumor microbiome cancer microbiome

integration integration

periodontitis periodontitis

biofilm formation biofilm formation

chimeric antigen receptor chimeric antigen receptor

acellular dermal matrices acellular dermal matrices

ecology ecology

adenomas adenomas

abscess abscess

5 fluorouracil 5 fluorouracil

2 sides 2 sides

5 fluorouracil 5 fluorouracil induced intestinal mucositis

augmentation augmentation

anaerobic bacteria anaerobic bacteria

adenomucinosis adenomucinosis

antigen delivery antigen delivery

braf mutation braf mutation

bacterial biofilms bacterial biofilms

diagnosis diagnosis

gut microbiome human gut microbiota

unifrac unifrac

bacterial communities bacterial communities

carcinomatosis carcinomatosis

16s rna sequencing 16s ribosomal rna

bile duct bile duct

coleys toxins coleys toxins

epstein barr virus epstein barr virus

cystic fibrosis cystic fibrosis

absence absence

cancers cancers

colonic mucosa colonic mucosa

bitter receptor bitter receptor
